# Supplementary material for: Effects of Surface IR783 Density on the In Vivo Behavior and Imaging Performance of Liposomes
Source: Pharmaceutics. 2024 May 30;16(6):744. doi: 10.3390/pharmaceutics16060744 (PMC11206891; doi:10.3390/pharmaceutics16060744)
Supplement: Supplementary file 1 [file pharmaceutics-16-00744-s001.zip › File S1. Figures S1-S3.pdf]

Supplementary Information

Effects of Surface IR783 Density on the In Vivo Behavior and Imaging Performance of Liposomes

Qianqian Long [1,†], Xinmin Zhao [1,†], Lili Gao [2], Mengyuan Liu [1], Feng Pan [1], Xihui Gao [3], Changyou Zhan [3], Yang Chen [4], Jialei Wang [1,*] and Jun Qian [1,*]

[1] School of Pharmacy, Department of Thoracic Medical Oncology, Fudan University Shanghai Cancer Center, Shanghai Medical College, Fudan University, Shanghai 200032, China
[2] Department of Pathology, Pudong New Area People's Hospital, Shanghai 201299, China
[3] School of Basic Medical Sciences, Fudan University, Shanghai 200032, China
[4] Key Laboratory of Separation Science for Analytical Chemistry, Dalian Institute of Chemical Physics, Chinese Academy of Sciences, Dalian 116023, China
* Correspondence: wangjialei@shca.org.cn (J.W.); qianjun@fudan.edu.cn (J.Q.)
† These authors contributed equally to this work.

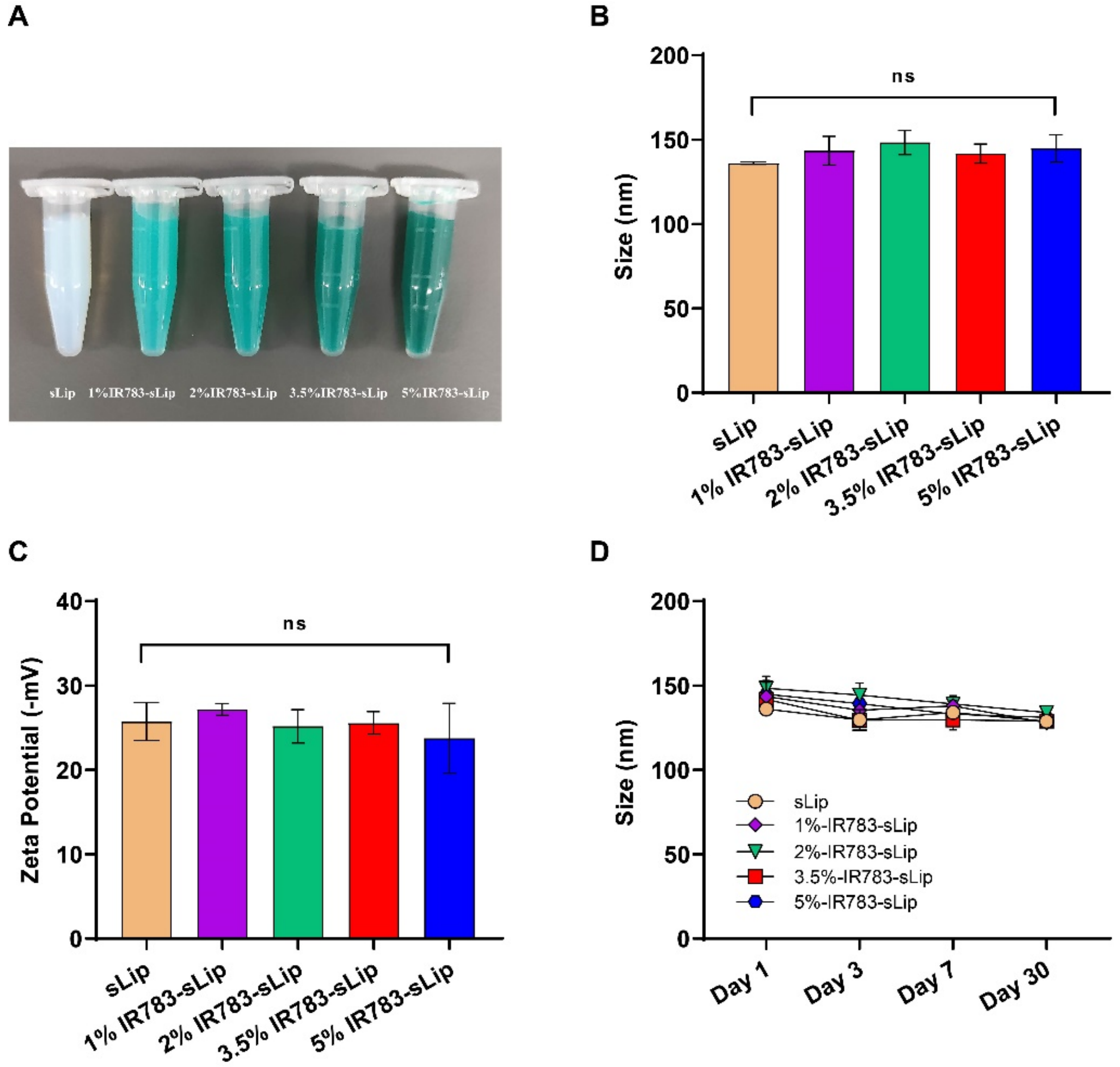

Figure S1. Characterization of IR783-sLip and sLip. The (A) optical photo (B) size, (C) zeta potential, and (D) stability of liposomes diluted 50 times with doubly distilled water.

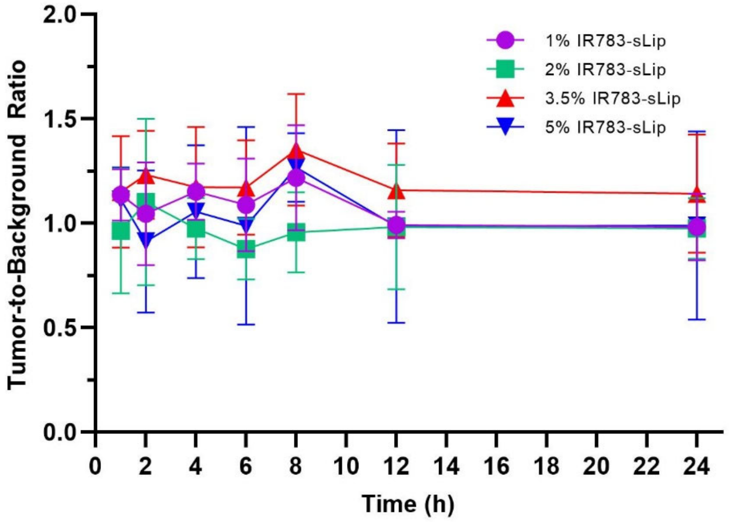

Figure S2. Change in the tumor-to-background ratio (TBR) over time. Plots of TBR values were calculated using the average fluorescence intensity of the tumor region of interest (ROI) divided by an average value of three independent background regions as measured by LivingImage software.

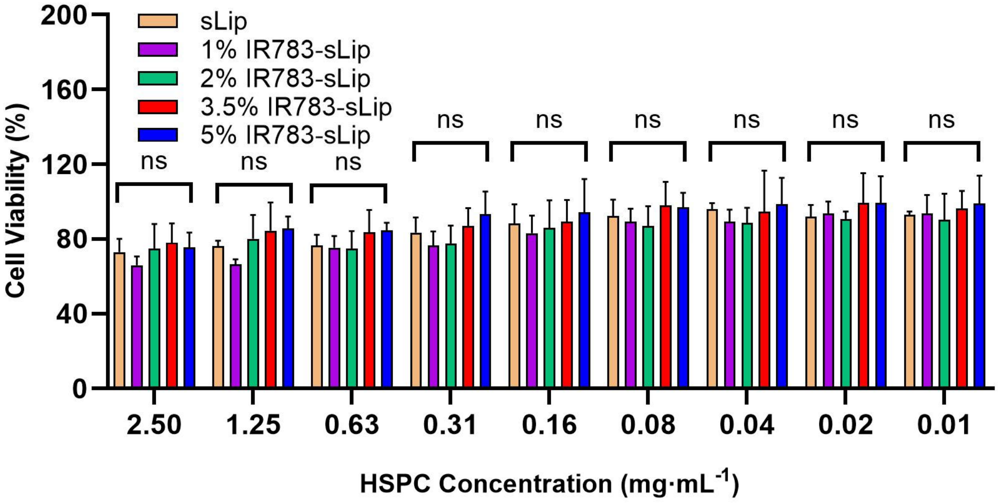

Figure S3. Cell viability of HUVEC cells incubated with four IR783-sLip for 72 h (n=3). The bars represent mean ± SD. Statistical significance was assessed by analysis of two-way ANOVA; ns, no significance.
