# Supplementary material for: Effects of Surface IR783 Density on the In Vivo Behavior and Imaging Performance of Liposomes
Source: Pharmaceutics. 2024 May 30;16(6):744. doi: 10.3390/pharmaceutics16060744 (PMC11206891; doi:10.3390/pharmaceutics16060744)
Supplement: Supplementary file 1 [file pharmaceutics-16-00744-s001.zip › File S2. Figure S4-original image+Table S1-revision.pdf]

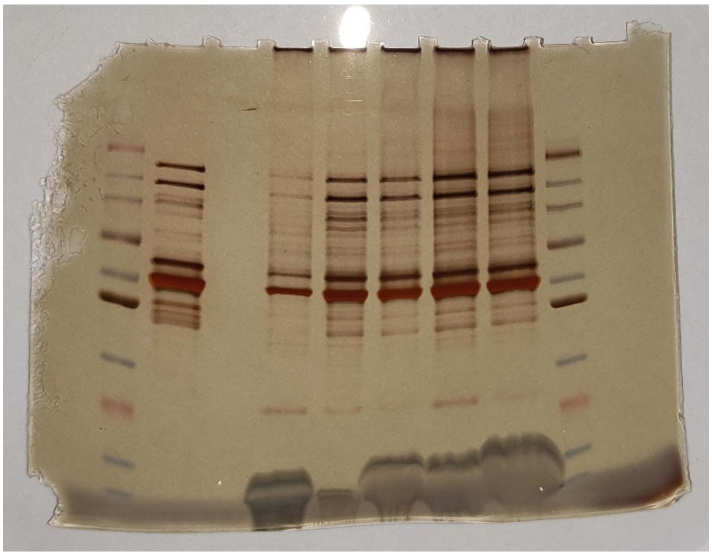

Figure S4a. The full-length gel image of Figure 5A. Lanes from left to right: Marker, Serum, sLip, 1% R783-sLip, 2% IR783-sLip, 3.5% IR783-sLip, 5% IR783-sLip, Marker.

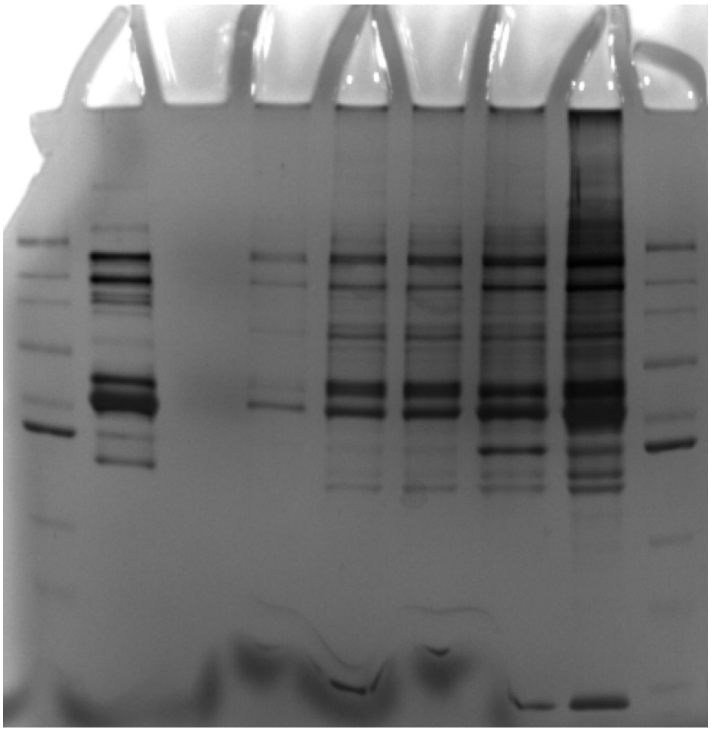

Figure S4b. The full-length gel image of Figure 5B. Lanes from left to right: Marker, Serum, sLip, 1% R783-sLip, 2% IR783-sLip, 3.5% IR783-sLip, 5% IR783-sLip, Marker.

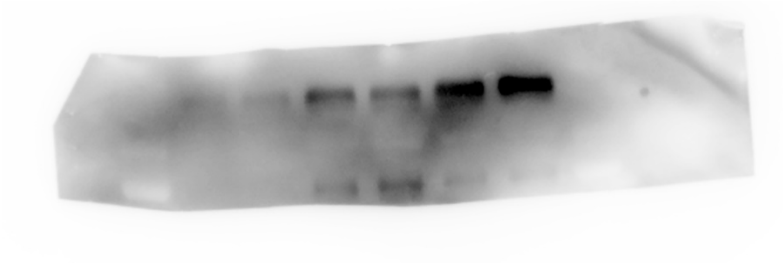

Figure S4c. The full-length lot image of figure 5C (i). Lanes from left to right: Marker, Serum, sLip, 1% R783-sLip, 2% IR783-sLip, 3.5% IR783-sLip, 5% IR783-sLip, Marker.

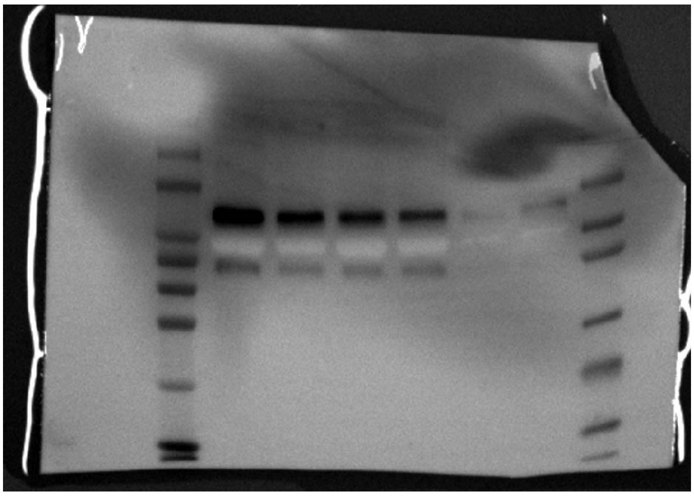

Figure S4d. The full-length lot image of figure 5C (ii). Lanes from left to right: Marker, Serum, sLip, 1% R783-sLip, 2% IR783-sLip, 3.5% IR783-sLip, 5% IR783-sLip, Marker.

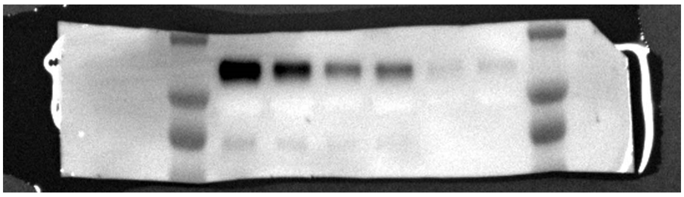

Figure S4e. The full-length lot image of figure 5C (iii). Lanes from left to right: Marker, 5% IR783-sLip, 3.5% IR783-sLip, 2% IR783-sLip, 1% IR783-sLip, sLip, Serum, Marker.

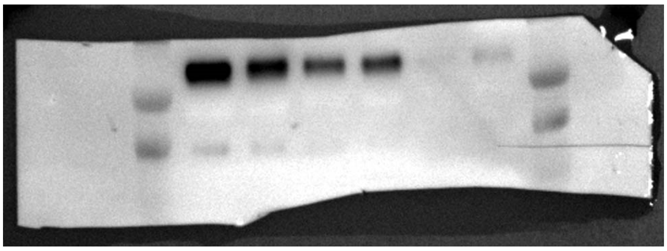

Figure S4f. The full-length lot image of figure 5C (iv). Lanes from left to right: Marker, 5% IR783-sLip, 3.5% IR783-sLip, 2% IR783-sLip, 1% IR783-sLip, sLip, Serum, Marker.

Table S1. Densitometry readings/intensity ratio of each band in Figure S4c-f and Figure 5C.

	Lanes	In vitro	In vivo		
		Figure S4c	Figure S4d	Figure S4e	Figure S4f
Intensity	5% IR783-sLip	27611.075	28464.903	27770.539	28610.953
	3.5% IR783-sLip	20686.953	20904.711	20950.66	20988.953
	2% IR783-sLip	10306.589	17115.296	10420.468	14161.711
	1% IR783-sLip	11283.347	14075.782	11323.882	15135.953
	sLip	1685.175	1782.326	1598.811	1469.397
	Serum	2714.397	4041.64	2774.933	3500.761
Normalized	5% IR783-sLip		7.042909067	10.0076431	8.172781
	3.5% IR783-sLip		5.172333756	7.54996967	5.99554011
	2% IR783-sLip		4.234740353	3.75521427	4.04532357
	1% IR783-sLip		3.48269069	4.08077673	4.32361792
	sLip		0.440990786	0.57616202	0.41973645
	Serum		1	1	1
